# Supplementary material for: Causal Relationship between Aging and Anorexia Nervosa: A White-Matter-Microstructure-Mediated Mendelian Randomization Analysis
Source: Biomedicines. 2024 Aug 16;12(8):1874. doi: 10.3390/biomedicines12081874 (PMC11351342; doi:10.3390/biomedicines12081874)
Supplement: Supplementary file 1 [file biomedicines-12-01874-s001.zip › Supplementary Figure/Figure S8/Leaveoneout plot of MR analysis for TL to ubm-b-1552.pdf]

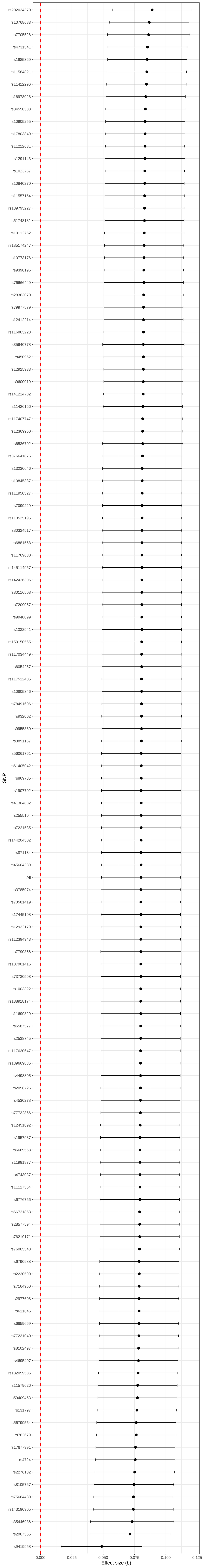

SNP
rs202034370
rs10768683
rs7705526
rs4731541
rs1985369
rs11584821
rs11412296
rs16978028
rs34550383
rs10905255
rs17803849
rs11212631
rs1291143
rs1023767
rs10840270
rs11557154
rs139795227
rs61748181
rs10112752
rs185174247
rs10773176
rs9398196
rs76666449
rs28363070
rs79977579
rs12412214
rs116863223
rs35640778
rs450962
rs12925933
rs9600019
rs141214782
rs11426156
rs117407747
rs12369950
rs6536702
rs376641875
rs13230646
rs10845387
rs111950327
rs7099229
rs113525195
rs80324517
rs6881568
rs11769630
rs145114957
rs142426306
rs80116508
rs7209057
rs9940099
rs1332941
rs150150565
rs117034449
rs6054257
rs117512405
rs10805346
rs78491606
rs932002
rs9955360
rs3891167
rs56061761
rs61405042
rs869785
rs1907702
rs41304832
rs2555104
rs7221585
rs144204502
rs871134
rs45604339
All
rs3785074
rs73581419
rs17445108
rs12932179
rs112394943
rs7790856
rs137901416
rs73730598
rs1003322
rs188918174
rs11699829
rs6587577
rs2538745
rs117630647
rs139669835
rs4498805
rs2056726
rs4530278
rs77732866
rs12451892
rs1957937
rs6669563
rs11991877
rs4743037
rs11117354
rs6776756
rs66731853
rs28577594
rs76219171
rs76065543
rs6790988
rs2230590
rs7164950
rs2977608
rs611646
rs6659669
rs77231040
rs8102497
rs4695407
rs182059586
rs11579626
rs59409453
rs131797
rs56799554
rs762679
rs17677991
rs4724
rs2276182
rs8105767
rs75664430
rs143190905
rs35446936
rs2967355
rs9419958
0.000
0.025
0.050
0.075
0.100
0.125
Effect size (b)
